# The life-span trajectory of visual perception of 3D objects

**DOI:** 10.1038/s41598-017-11406-7

**Published:** 2017-09-08

**Authors:** Erez Freud, Marlene Behrmann

**Affiliations:** Department of Psychology and the Center of Neural Basis of Cognition, Carnegie Mellon University, Pittsburgh, Pennsylvania United States

## Abstract

Deriving a 3D structural representation of an object from its 2D input is one of the great challenges for the visual system and yet, this type of representation is critical for the successful recognition of and interaction with objects. Perhaps reflecting the importance of this computation, infants have some sensitivity to 3D structural information, and this sensitivity is, at least, partially preserved in the elderly population. To map precisely the life-span trajectory of this key visual computation, in a series of experiments, we compared the performance of observers from ages 4 to 86 years on displays of objects that either obey or violate possible 3D structure. The major findings indicate that the ability to derive fine-grained 3D object representations emerges after a prolonged developmental trajectory and is contingent on the explicit processing of depth information even in late childhood. In contrast, the sensitivity to object 3D structure remains stable even through late adulthood despite the overall reduction in perceptual competence. Together, these results uncover the developmental process of an important perceptual skill, revealing that the initial, coarse sensitivity to 3D information is refined, automatized and retained over the lifespan.

## Introduction

One of the major challenges confronting the visual system is the need to transform the two-dimensional (2D) retinal information into a precise three-dimensional (3D) representation of the visual input. This 3D representation is critical for successful object recognition and visually guided interactions with objects. For example, the identification of a pair of scissors seen from a particular vantage point depends on the ability of the observer to interpret the 3D structure of the scissors. This interpretation supports the recognition of the scissors across a multitude of transformations, and permits the planning of the appropriate action. Remarkably, the visual system solves this computational challenge many thousands of times in the course of a day, and generates stable and accurate 3D representations of the world rapidly and effortlessly^1^.

Given the importance of 3D visual representations for perception and action, considerable research has been devoted to understanding the life-span trajectory of the ability to derive such a representation. Studies have shown that infants are sensitive to depth cues (for a recent review see ref. 2), and that this sensitivity might subserve the representation of object 3D structure^3, 4^. However, most of these studies assess the derivation of a coarse 3D volume in which case the infant need only derive a limited representation of the object 3D structure. Whether and when young children are able to represent a 3D shape, which encompasses the detailed description of the object’s planes and axes, and which is then engaged automatically in a task-independent fashion^5, 6^ as is the case in adulthood, remains to be determined. Although this particular question has not been addressed previously, empirical evidence that tracks the emergence of mature, complex visual perception reveals that visual computations are immature and evince a slow developmental trajectory: for example, both the ability to integrate contours over spatial distance and the ability to abstract viewpoint-invariant representations of objects are immature in young children, reaching maturity at approximately 14 years of age^7–9^. Whether this same age-related pattern holds for 3D visual derivation remains to be determined.

At the other end of the age continuum (for a recent review see, ref. 10), the consensus is that, while some aspects of 3D perception, mainly related to motion, are impaired in late adulthood, other aspects are preserved^11–13^. For example, Norman and colleagues found that older and young adults performed equally well when judging whether two depicted 3D objects, presented in different angular offsets, were instances of the same object^12^. Examining the ability to derive coherent 3D structure across the life span will therefore shed further light on the primacy of key visual computations.

**Figure 1:**
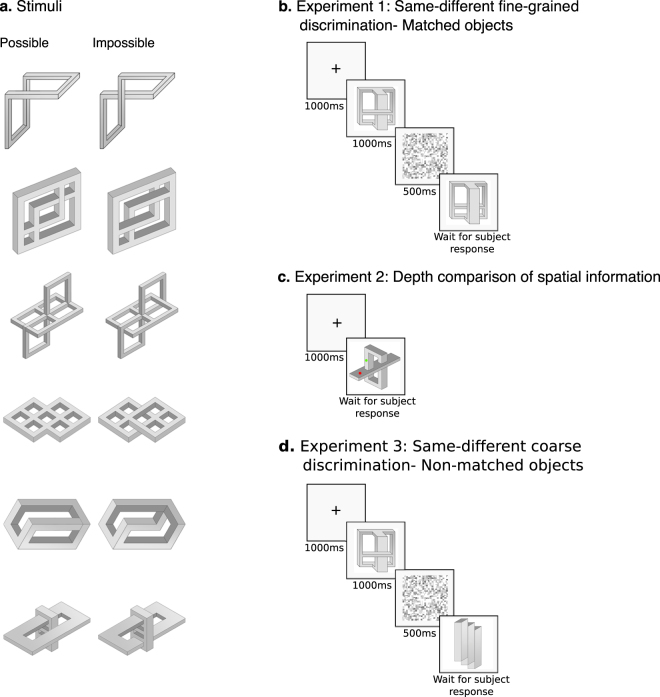
Stimuli and experimental design (**a**) Examples of possible (left panel) and matched impossible objects (right panel). Note that there are minimal physical differences between the two object types, but the perceptual experience in viewing these two object sets is substantially different. (**b**) Experiment 1: Same-different discrimination of matched objects: Participants performed same/different discriminations on sequentially presented objects, which were physically highly similar even in the ‘different’ trials. (**c**) Experiment 2: Comparison of depth information: Participants judged which dot is closer in depth - the red or the green dot. (**d**) Experiment 3: Same-different discrimination of non-matched objects: Participants performed same/different classifications on sequentially presented objects, which were physically different.

Here, we examine age-related changes in the ability to derive 3D structural representations by presenting visual images of objects that obey (possible) or violate (impossible) 3D geometry to participants aged from 4 to 86 years (Fig. 1a). The two classes of objects require precise computation of the edges and angles^5, 14^ and share a high-degree of visual similarity. In contrast with possible objects, impossible objects contain inconsistencies between global and local structure: while the local cues are valid, the resulting 3D global structure is incoherent and does not respect the legality of the structure of real-world objects. Better performance on displays of possible over impossible objects reveals sensitivity to the laws that govern 3D representations. Furthermore, the perceptual advantage of possible over impossible objects even when participants are not explicitly required to derive this structural information, indicates that the 3D information is automatically and obligatorily derived^5^.

Evidence of sensitivity to object possibility by infants^15^ has been uncovered but because, only a single exemplar (a possible versus an impossible cube) was used in this study, the infants’ preferential looking may have reflected sensitivity to 2D novel line junctions embedded in the impossible (but not possible) cube rather than the differential perception of the two displays^16^. Indeed, a similar study reported limited sensitivity to object possibility in late childhood (6–11 years old), indicating that shape integration is immature in children^17^. Note, however, that this latter study required participants to classify the possibility/impossibility of the displayed objects, and such a judgment may engage immature non-perceptual processes rather than reflecting perceptual skills per se. Definitive conclusions about 3D perception in later adulthood have also not been reached, although possible/impossible stimuli have been used previously to reveal impairments in long-term memory encoding of structural information^18^.

In the present study, we compared the performance of five groups of participants on three different experiments, all involving images of possible and impossible objects. These stimuli and procedures have successfully uncovered adult observers’ sensitivity to structural information previously^19^. In all of the experiments, a large number of displays were used and perceptual demands were varied. Also, to circumvent the need for overt classification of the objects’ status (possible versus impossible), object status is always orthogonal to the demands of the task.

## Results

### Experiment 1- Same-different discriminations-matched objects

In Experiment 1, participants decided whether two possible and/or impossible objects displayed sequentially on a computer screen were the same or different (see Fig. 1b and Methods for details). Half the trials were of the same objects and the remaining half contained objects that differed by a small local feature, requiring precise representation of the objects for correct performance. Previous findings have shown that if the first object is possible, performance is better than if the first object is impossible (a coherent representation could not be generated) even when the task does not require the observer to label the object status overtly^14, 19, 20^. The failure to show this signature first object advantage would indicate perceptual immaturity in children and decrement of this ability in later adulthood.

**Figure 2:**
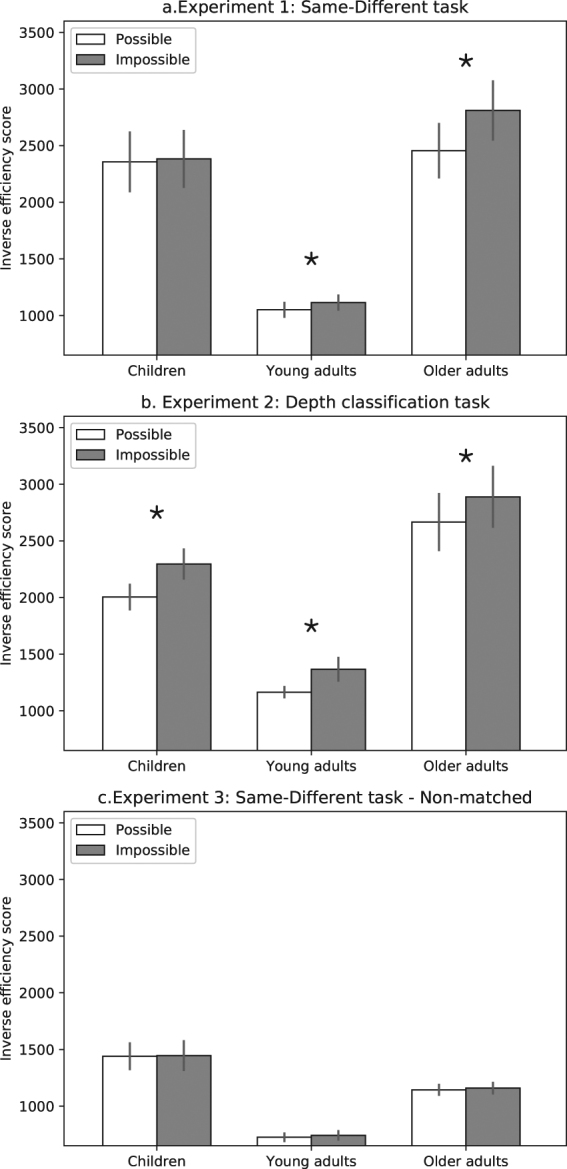
Results. (**a**) Experiment 1: Children and older adults performed poorly overall relative to young adults. While younger and older adults exhibited sensitivity to structural information with better performance for trials in which the first object was a possible object, children did not show this effect. Trial type (same/different) did not interact with first object type and, therefore, the graph is collapsed across this factor. (**b**) Experiment 2: Children and older adults, performed poorly relative to young adults. In contrast with Experiment 1, all groups were sensitive to structural information with better performance for possible than impossible objects. (**c**) Children and older adults performed poorly overall relative to young adults. All groups exhibited similar performance for possible and impossible objects. Note that the analyses were conducted on transformed data (see Methods for details) but that, for the sake of clarity, data are presented in raw units. Error bars represent the standard error of the mean for each condition and asterisks indicate significant difference between the two object categories.

A repeated measures ANOVA with group (children, young adults and older adults; between subjects), object type (possible/impossible) and trial type (same/different) as independent variables, and inverse efficiency scores^21^ (IE; mean RT/proportion of correct trials) as the dependent measure (taking both accuracy and speed into account), revealed that children and older adults performed significantly more poorly than young adults [main effect of group: F_(2,50)_ = 29.04, η_p_^2^ = 0.53, p < 0.05, planned comparison: F_(2,50)_ = 57.4, p < 0.05; Fig. 2a]. More importantly, the ANOVA revealed a significant two-way interaction between group and object type [F_(2,50)_ = 3.13, η_p_^2^ = 0.11, p = 0.05]. As in previous studies^14, 19^, young adults evinced sensitivity to 3D structural information, with better performance for the trials primed by possible than impossible objects [F_(1,50)_ = 4.17, p < 0.05, mean IE difference = 64]. While older adults do show such a pattern, indicating sensitivity to structural information [F_(1,50)_ = 16.59, p < 0.05, mean IE difference = 355], this pattern was not present for the children [F_(1,50)_ < 1, mean IE difference = 26] (Fig. 2a). An additional main effect was found for trial type [F_(1,50)_ = 29, η_p_^2^ = 0.36, p < 0.05], with better performance for the “same” than “different” trials, but more importantly, no interactions of trial type with object type nor with group were found [Fs < 1]. Analysis of the accuracy score (d′) revealed only a main effect of group [F_(1,50)_ = 13.1, η_p_^2^ = 0.33, p < 0.05] with no interaction between group and object type [F_(1,50)_ = 1.2, η_p_^2^ = 0.04, p > 0.25]. Notably, when RT was used as the main dependent variable, a similar pattern to the IE scores analysis was found [Interaction effect: F_(1,50)_ = 2.45, η_p_^2^ = 0.09, p = 0.09] with both old adults [F_(1,50)_ = 9.18, p < 0.05] and young adults [F_(1,50)_ = 8.37, p < 0.05] exhibiting faster RTs for possible objects compared with impossible objects. No difference between object type was observed for the children’s group [F_(1,50)_ < 1] (see Table 1).

**Table 1:** Average (and standard error) Sensitivity (d′) and RT (ms) as function of Experiment and group.

Group		Same-different (matched)		Depth task		Same-different (non-matched)	
		Possible	Impossible	Possible	Impossible	Possible	Impossible
Children	d′	2.42 (0.18)	2.31 (0.2)	3.53 (0.18)	3.22 (0.16)	3.64 (0.1)	3.76 (0.12)
	RT	1757 (136)	1743 (115)	1855 (113)	2054 (118)	1336 (109)	1364 (128)
Young adults	d′	3.27 (0.18)	3.26 (0.18)	4.1 (0.12)	3.7 (0.14)	3.94 (0.1)	4.03 (0.09)
	RT	923 (55)	986 (60)	1117 (55)	1263 (96)	694 (42)	711 (42)
Old adults	d′	2.11 (0.19)	1.82 (0.19)	3.53 (0.22)	3.24 (0.19)	4.22 (0.1)	4.17 (0.11)
	RT	1698 (110)	1861 (144)	2421 (208)	2527 (215)	1144 (58)	1146 (56)
Children (instructed depth processing)	d′	2.56 (0.16)	2.18 (0.18)	3.23 (0.26)	3.1 (0.25)	3.16 (0.19)	3.34 (0.14)
	RT	1663 (108)	1712 (106)	1757 (91)	1888 (122)	1420 (79)	1390 (63)

These findings offer novel evidence for the immaturity of 3D structural representations even in later childhood, under conditions in which stimuli are similar to each other and precise perceptual encoding is required. This slow maturation is supported by recent findings that viewpoint invariant object representation, which also relies on a fine-grained description of 3D information, was both neurally and behaviorally immature in childhood^9^. Older adults, although performing more poorly overall than the younger adults, retained sensitivity to the legality of structural information, even when it was irrelevant to the task. The preserved sensitivity of older adults to this type of information is consistent with previous studies^11, 12^, and indicates that the processing of object 3D structure (based on monocular, stationary depth cues) in older adults is largely preserved and deployed automatically.

Importantly, the lack of sensitivity to 3D information in the children could not be attributed to a floor effect, because their average accuracy scores were substantially above chance (mean d′: 2.42 for possible and 2.31 for impossible objects), and the older adults, who were sensitive to structural information, had even lower accuracy scores (mean d′:2.11 for possible and 1.82 for impossible objects, see Table 1).

### Experiment 2 – Depth comparisons

The lack of sensitivity of children to structural information might be accounted for by two explanations: the first is that children simply are unable to derive a fine-grained representation of structural information. Alternatively, children may have the capability to derive 3D structural representations, but these representations are not engaged automatically, as is the case in adulthood. To adjudicate between these alternatives, Experiment 2 utilized a task that specifically instructed the participants to process the 3D information of the image (see Fig. 1c) by judging which of two colored dots in the display was located closer in depth^22^. Better performance for structurally coherent objects relative to the impossible objects is indicative of underlying sensitivity of the visual system to 3D structural information. If children can derive 3D representations but differ from adults in the automaticity of this process, in contrast to Experiment 1, we predict that children would be sensitive to structural information in Experiment 2. Note again that in all experiments, whether an object is possible or not is orthogonal to the task at hand.

As plotted in Fig. 2b, a main effect of object type was found [F_(1,50)_ = 34.25, η_p_^2^ = 0.4, p < 0.05], and there was no interaction with group [F_(2,50)_ < 1, η_p_^2^ = 0.03]. Sensitivity to structural information, with better dot depth classification located on possible than on impossible objects, was equally evident in the young [F_(1,50)_ = 15.8, p < 0.05, mean IE difference = 201] and older adults [F_(1,50)_ = 5.03, p < 0.05, mean IE difference = 222], as well as in children [F_(1,50)_ = 15.5, p < 0.05, mean IE difference = 291]. As in Experiment 1, this sensitivity to the structure of the object was observed despite the relatively poorer overall performance in children and in older adults (see y-axis) [F_(2,50)_ = 28.13, η_p_^2^ = 0.52, p < 0.05; planned comparison: F_(2,50)_ = 57.4]. The sensitivity to object type across all groups was also found when accuracy [F_(1,50)_ = 17.7, η_p_^2^ = 0.26, p < 0.05, Table 1] and RT [F_(1,50)_ = 25.29, η_p_^2^ = 0.33, p < 0.05, Table 1] were tested separately, with no interaction between group and sensitivity to object type [Fs < 1].

Next, to compare between the two experiments directly, an additional ANOVA with IE scores as the dependent variable, experiment, object type and group as independent variable was conducted. This analysis revealed a three-way interaction that confirms the differential pattern of results obtained in Experiment 1 (i.e. insensitivity of children relative to the other groups) in comparison with Experiment 2 (i.e. sensitivity of children, similar to the other groups) [F_(2,50)_ = 4.07, η_p_^2^ = 0.14, p < 0.05].

### Experiment 3- Same-different discrimination-non-matched objects

The results presented so far have shown that sensitivity to object 3D structure is preserved in older adults, but is immature in young children and relies on explicit depth processing. However, the observed differences between the two experiments could be related to the different task demands, and not to the degree to which fine-grained depth processing was needed. To rule out this alternative account, the participants performed a same-different task, but unlike Experiment 1, stimuli in the ‘different’ trials were obviously different (Fig. 1d). Hence, this task could be performed based on coarse differences between objects and did not require fine-grained processing of the 3D properties of the stimuli.

As plotted in Fig. 2c, a main effect for group was found [F_(2,50)_ = 25.15, η_p_^2^ = 0.5, p < 0.05] and planned comparison confirmed that young adults performed better than the children and older adults [F_(2,50)_ = 46.26, p < 0.05]. In contrast to Experiment 1 and 2, no effect of object type and no interaction between object type and group were found [Fs < 1], and planned comparisons showed that the performance in all groups was similar for the two object categories [Fs < 1]. Similar effects were observed in terms of RT and d′, with no effects of object type [Fs < 1, Table 1], no interaction between object type and group [d′: F < 1; RT: F_(2,50)_ = 1.13, η_p_^2^ = 0.04, p > 0.3] and a main effect for group [d′: F_(2,50)_ = 23.6, η_p_^2^ = 0.48, p < 0.05; RT: F_(2,50)_ = 6.4, η_p_^2^ = 0.2, p < 0.05].

Finally, the sensitivity to object type (i.e., the lower IE scores for possible objects) across the three experiments was compared. Given the differential sensitivity of the children group in Experiment 1, this analysis was conducted separately for this group. These analyses revealed an interaction between Experiment and object type (adults: F_(2,70)_ = 20.62, η_p_^2^ = 0.37, p < 0.05, children: F_(2,34)_ = 4.43, η_p_^2^ = 0.2, p < 0.05), reflecting the lack of sensitivity to object type in Experiment 3, and the sensitivity to object type in Experiment 1 (old and young adults) and Experiment 2 (all groups). Note that similar interaction was found when all groups were included in the same ANOVA (F_(2,100)_ = 7.84, η_p_^2^ = 0.13, p < 0.05).

Hence, Experiment 3 suggests that the better performance for possible over impossible objects, observed in Experiment 1, are not the product of decision making or response demands, but, rather are related to 3D fine-grained representations which were required in Experiment 1 and 2, but not in Experiment 3. Notably, it is still plausible that the 3D information was automatically processed even in Experiment 3^6^, but that the lack of sensitivity to object type suggests that this processing was coarse and limited. Finally, the equivalent performance for the two object types in this last experiment, might be interpreted as a result of a ceiling effect; however, similar high sensitivity scores were also observed in Experiment 2 and sensitivity to object type was still evident in this experiment. Moreover, a between-groups main effect was found in Experiment 3, further suggesting that the performance of at least the older participants and children groups were not at ceiling.

Two main conclusions emerge from the experiments presented above. First, despite the infants’ sensitivity to 3D information observed in previous studies^2–4^, here, children showed reduced sensitivity (i.e., reduced differences in response to possible versus impossible objects) to fine-grained 3D structural information compared with young and older adults. Intriguingly, even patients with visual agnosia, who have a profound impairment in object recognition, still exhibit sensitivity to structural information in the task employed in Experiment 1^19^. When required to engage in explicit processing of depth information to solve the task, however, children exhibited preserved sensitivity to object type (Experiment 2). This finding suggests that, despite the immaturity of object 3D structure processing in children (aged 7.5–13 years old in that experiment), fine-grained descriptions of the 3D structure can be derived when children are explicitly instructed to take depth information into account.

Second, despite a general reduction in perceptual abilities, older adults maintained sensitivity to object 3D structural information. These results are compatible with previous investigations^11, 12^, and extend the findings by showing that this preserved sensitivity is obligatorily evoked (even when the task does not require consideration of the 3D structure of the object) and can support fine-grained representations of object type. The observed perceptual sensitivity contrasts with the reported impairment in which memory-based representation of structural information and contextual knowledge about object type, is compromised in older adults^18^.

### Engagement with depth processing enhanced depth sensitivity in children

If it is indeed the case that young children are able to represent 3D structural information but that, in the immature system, left to its own devices, this ability is not automatically engaged, then we might expect that children instructed specifically to use this information might show the advantage for possible over impossible objects in the fine-grained same-different task used above (Experiment 1). To explore this, we recruited a new group of children to complete Experiments 1 and 2. To emphasize the processing of object 3D structural information, we (a) specifically instructed the children to use depth information for their judgments and (b) altered the order of the experiments so that the depth comparison task (Experiment 2 that explicitly engages 3D representations) is completed before the task of same/different object discrimination. We compared the performance of this ‘instructed depth’ group with the data from the previous ‘non-instructed’ group of children.

**Figure 3:**
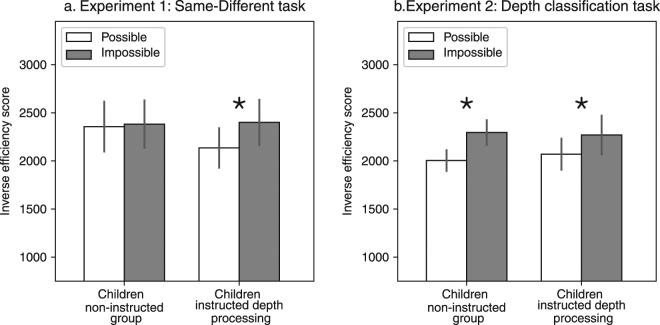
Results of the comparison of the two groups of children. (**a**) Experiment 1: When instructed to process depth information explicitly, and when this experiment was completed as the second experiment, children in the instructed depth group demonstrated sensitivity to structural information. On the other hand, children not instructed to attend to depth were not sensitive to structural information (data repeated from Fig. 2). Note that the overall level of performance was similar between the two groups. Trial type (same/different) did not interact with object type and, therefore, the graph is collapsed across this factor. (**b**) Experiment 2: The two groups performed equivalently on this task in which depth information is explicitly taken into account. Error bars represent the standard error of the mean for each condition and asterisks indicate significant difference between the two object categories. The key finding is that the instructed depth children who performed Experiment 2 before Experiment 1 now showed the advantage for possible over impossible objects in Experiment 1.

As expected, in Experiment 2 (depth task), a main effect of object type was found [F_(1,31)_ = 18.56, η_p_^2^ = 0.37, p < 0.05], with no interaction with group (non-instructed/instructed) [F_(1,31)_ = 1.19, η_p_^2^ = 0.03, p > 0.25], and planned comparisons validated that children in the instructed depth processing groups were as sensitive to structural information as the non-instructed group [F_(1,31)_ = 13, p < 0.05, mean difference = 200] (Fig. 3b). The analyses of accuracy and RT data also revealed main effects of object type [d′ - F_(1,31)_ = 6.39, η_p_^2^ = 0.17, p < 0.05; RT - F_(1,31)_ = 14.56, η_p_^2^ = 0.31, p < 0.05] with no interaction with group [d′ - F_(1,31)_ = 1.11, η_p_^2^ = 0.03, p > 0.2; RT – F_(1,31)_ < 1].

The critical comparison between the instructed and non-instructed children is in Experiment 1 (same-different task). Repeated measures ANOVA with group, object type and trial type (same/different) revealed a main effect of object type [F_(1,31)_ = 7.31, η_p_^2^ = 0.19, p < 0.05], but this effect was qualified by a two-way interaction between group and object type [F_(1,31)_ = 4.93, η_p_^2^ = 0.13, p < 0.05]. In contrast with the non-instructed children, the children in the instructed depth group revealed sensitivity to object structural information [F_(1,31)_ = 13, p < 0.05, mean difference = 265] (Fig. 3a). A main effect for trial type was found [F_(1,31)_ = 29, η_p_^2^ = 0.49, p < 0.05], and there were no interactions with any other factor [Fs < 1]. Thus, by simply manipulating the instruction and order of experiments, sensitivity to 3D information became evident in children. The interaction between group and object type was not found when d′ and RT served as dependent variables [d′ - F_(1,31)_ = 1.91, η_p_^2^ = 0.06, p = 0.17; RT – F_(1,31)_ < 1]. However, simple comparisons showed that accuracy was greater for possible object over impossible objects for the instructed depth group [F_(1,31)_ = 8.53, p < 0.05] and not for the first children group [F_(1,31)_ = 1.31, p > 0.25].

To compare between the two experiments, an ANOVA with group, experiment and object type as independent variables revealed a significant three-way interaction [F_(1,31)_ = 6.05, η_p_^2^ = 0.16, p < 0.05], and planned comparisons confirmed that this interaction stemmed from differences in the effect of object type on the two children groups in Experiment 1 [F_(1,31)_ = 4.92, p < 0.05], but no difference in the effect of object type in Experiment 2 [F_(1,31)_ = 1.18, p > 0.25]. Moreover, no main effect for group was found [F_(1,31)_ < 1] and this was also true in terms of accuracy [F_(1,31)_ < 1]. Finally, a trend of a three-way interaction was also found when d′ served as the dependent variable [F_(1,31)_ = 3.35, η_p_^2^ = 0.1, p = 0.07]. That the sensitivity to fine-grained 3D information is evident when depth information is engaged suggests that children are indeed capable of representing 3D information but that this ability is not evoked under standard perceptual conditions. Note that the instruction to refer to depth information did not facilitate overall perceptual performance, but, rather, selectively increased the sensitivity to structural information in the children aged 7.5–13 years of age.

### Explicit processing does not support structural information sensitivity in younger children

These findings indicate a certain amount of competence in the young children, which does not manifest in their performance unless specifically instructed. Of course, this raises the question as to whether this latent competence is present at even younger ages than those tested thus far. To address this question, in this last experiment, we examined whether younger children (aged 4–6.5 years old) are able to interpret fine-grained depth information when explicitly directed to take depth into account. We explored this in the context of the red-green dot depth comparison task. To ensure that any observed failure is not a function of fatigue or non-compliance, we shortened the original experiment (now only 42 trials).

**Figure 4:**
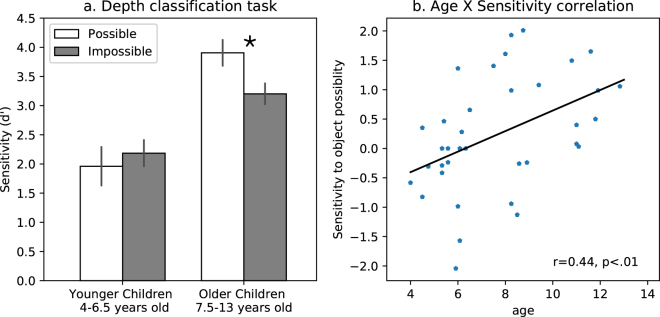
Results of the comparison of younger and older children. (**a**) Comparison between younger (4–6.5 years old) and older, non-instructed children (7.5–13 years old) in the depth classification task. The data from the older children are based on a subset of the stimuli (first 42 trials). Greater sensitivity to possible than impossible objects was observed only in the older children. (**b**) Correlation between age and sensitivity to object type. Sensitivity to object type increased as function of age.

The analysis was focused on accuracy (d′) data, as these young children responded verbally and their RT data were therefore not comparable to that of the older children. ANOVA with group (older children-non-instructed group (7.5–13 years old) and younger children (4–6.5 years old)) as a between-subjects factor and object type as a within-subjects factor revealed a significant interaction between object type and group [F_(1,35)_ = 10.63, η_p_^2^ = 0.23, p < 0.05]. Planned comparisons showed significantly better performance for possible over impossible objects in the older children [F_(1,35)_ = 11.9, p < 0.05], while no sensitivity was apparent in the younger children [F_(1,35)_ = 1.27, p > 0.25] (Fig. 4a). A main effect of group, with better performance for the older than younger children was found [F_(1,35)_ = 19, η_p_^2^ = 0.35, p < 0.01]. Importantly, in this experiment in which the use of depth information is part of the task requirements, young children still do not evince sensitivity to 3D possibility even though overall performance was above chance (d′ was well above 0 (chance level)), and only two out of 19 children exhibited an overall d′ that was lower than 0.3. Therefore, the absence of an advantage for the possible over impossible objects cannot be attributed to the misunderstanding of the instructions or the inability to perform the task more generally. Note that we equalized the number of stimuli presented to the groups based on two different methods: we extracted the responses of the older children for the first 42 trials, or, in the second method, we extracted the responses to the exact same stimuli from the older children’s’ data. The results presented here are based on the first 42 trials comparison, but similar results were observed for the same stimuli subset.

Finally, together, these two groups included 37 children of different ages (4–13 years old), and, therefore, we were able to explore whether sensitivity to object 3D structure (an advantage for possible over impossible objects) might be correlated with the age of the participants. To do so, for each participant, we first calculated a selectivity index (d′ possible – d′ impossible) in which positive values indicate that possible objects had better accuracy compared to impossible objects while negative values indicate the opposite. Next, we calculated the Pearson correlation of this index with participants’ age in years and months. This analysis revealed a significant correlation between these two variables [r = 0.44, t_(35)_ = 2.95 p < 0.01], such that the older a child, the greater the sensitivity to structural information (Fig. 4b), reflecting the developmental trajectory of sensitivity to 3D information.

Notably, at very young ages there was an unpredictable trend toward a negative sensitivity to object possibility (i.e., better performance for impossible objects; see left bars in Fig. 4a and bottom left of graph in Fig. 4b). One possibility is that this negative sensitivity might simply reflect noise in the data in the youngest children. However, if it is the case that the youngest children are more sensitive to impossible than to possible objects, it might suggest that sensitivity to structural information does not increase with age, but, rather, that, at young ages, a qualitatively different processing style is employed. For example, it plausible that young children adopt a more piecemeal processing style (which is easier for impossible objects that do not have a global coherent solution). This effect was not evident at the group level, perhaps because of the averaging of the participants, and could not explain the overall positive correlation between age and sensitivity to object possibility. The trend deserves further exploration to evaluate its reliability and significance.

## Discussion

Overall, the findings from the experiments described here provide novel evidence elucidating the life-span trajectory of the perception of 3D structural representations. We revealed that whereas young children (aged 4–6.5 years), as a group, were unable to compute a fine-grained depth description of an image from a 2D display, children aged roughly 7 years and older were able to do so. Intriguingly, however, even in these older children, left to their own devices, this structural information was not engaged unless specifically triggered top-down by explicit instructions. In sum, the perception of 3D object structure is not adult-like even in late childhood. In contrast, and notwithstanding the general decline of perceptual abilities, the sensitivity to 3D structural information was preserved in older adulthood.

Importantly, our experimental design enabled us to disentangle any reduction in overall perceptual competence from the sensitivity to structural information. Unsurprisingly, in light of previous studies showing a generalized slowing in older adults versus younger adults^23^ and in children, as well^24^, younger adults performed better in all experiments than did the other groups. This main effect of group, however, was dissociated from the differential sensitivity to structural information, as reflected by the advantage for possible over impossible objects in older adults but not in the children.

To capture both increased speed but also differences in accuracy across the groups, we primarily used inverse efficiency (IE) as the dependent measure and did so for a host of reasons. First, the utilization of IE^21^ as the main dependent variable is consistent with a large body of developmental and cognitive literature (e.g., refs 9, 25–27) and is considered to be particularly effective in situations in which different groups exhibit robust differences in accuracy (as evident in the present study)^28^. IE also has the potential to uncover effects that might not be easily observable when using just RT or accuracy scores. IE has, however, been criticized in the past and some have argued that it is not advisable to utilize IE when there is a speed-accuracy tradeoff^29^. Preliminary analysis revealed that in all experiments, there was a weak-moderate positive correlation between RT and error rate (Experiment 1 r = 0.54; Experiment 2: r = 0.1 Experiment 3 r = 0.25), providing no evidence for speed-accuracy tradeoff, which would be manifested as a negative correlation between RT and error rate.

Nonetheless, in addition to the IE, we also analyzed and presented the RT and accuracy data. For the comparisons between children and adults (i.e., Experiment 1), the RT analyses were largely consistent with the IE results. In particular, even though the interactions in RT between the group of children and the other groups (young and older adults) was only marginally significant, the simple comparisons replicated the results observed for the IE scores. The same holds for the comparisons between the children groups (i.e., instructed and non-instructed), in that the d′ simple comparisons showed a similar pattern to that observed when IE served as the dependent variable, even though the d′ interaction was not significant. Together, these additional analyses further suggest that the utilization of IE as the main dependent variable enabled us to compare between groups with seemingly different biases to reveal the developmental trajectory of the sensitivity to structural information.

Based on our findings, we have argued in favor of a change in perceptual competence over age. Age, however, is not necessarily the key factor in and of itself. It remains possible that some other visual, cognitive or developmental factor is the key that unlocks the perceptual skills. Age, here, serves only as a proxy and further research to explore the contribution of other possible factors would add greatly to the findings we have presented.

### The development of visual functions - from coarse to fine-grained representations

What plausible mechanisms might account both for the findings of early sensitivity to depth information observed in infant studies and the reduced sensitivity in young children reported here? One possible reconciliation of these seemingly contradictory findings is that the maturational sequence of the visual system depends on experience, and that more critical functions mature earlier^8^. Hence, early coarse sensitivity, which is crucial for perception and action, develops early, while more refined sensitivity emerges later in-life.

A similar coarse-to-fine profile is noted in age-related changes in other visual functions, such as contour integration^8^ and derivation of viewpoint-invariant^9^ representations of objects as mentioned in the introduction. Another domain in which a similar maturational trajectory is reported is that of face processing. Whereas newborns exhibit a looking preference for face-like over non-face like patterns^30, 31^, this bias may be based on coarse information such as spatial frequency or upright-heavy features and the representations derived are far from adult-like. Indeed, infants’ face sensitivity appears to rely on low-spatial-frequency information that provides a coarse description of the face^32^, and this ability may even be mediated by subcortical neural mechanisms^33^. Consistently, notwithstanding how essential face recognition is in day-to-day interactions, fine-grained face processing abilities develop slowly and reach maturity only in late adolescence^34^ or, as some have argued, even later, around 30 years of age^35^. Taken together, there may be a general principle that governs the emergence of complex visual pattern recognition in which an initial coarse ability suffices and then is followed by a coarse-to-fine trajectory that is relatively extended in time. This principle of ‘starting small’^36^ may cut across many domains of development including language and perception and the initial developmental restrictions may be critical for subsequent mastery in complex domains.

## Conclusions

In sum, by directly examining visual perception across the lifespan from age 4 to age 86, we have provided novel evidence for the preservation of 3D structural information representations in late adulthood alongside the slowly emerging skills in childhood. The initial perceptual abilities may suffice to bootstrap the system with subsequent mature skills emerging in a fine-grained but also automatic fashion. Once in place, these skills persistent even to older adulthood. Whether this signature of life-span development holds for other complex perceptual computations and whether this profile applies in other domains in which mastery of complex domains is required, remains to be investigated.

## Methods

### Participants

Data were analyzed from eighteen children (ages: 7.5–12.8 years, mean age: 9.68, SD: 1.66, 9 females), eighteen young (ages: 18.3–21.6 years, mean: 19.7, SD: 0.98, 9 females) and seventeen older adults (ages: 61–86, mean: 74.5, SD: 8.2, 12 females). An additional fifteen children (ages: 7.5–11.75 years old, mean: 9.32, SD: 1.19, 5 females) and nineteen young children (ages: 4–6.5 years old, mean: 5.44, SD: 0.75, 10 females) participated in the follow-up experiments.

Ten additional participants were excluded. The data from five participants (two young adults, one older adult and two older children) were not analyzed because the participants did not follow experimental instructions. Five additional participants (two older children and three older adults) were excluded as their accuracy was more than 2.5 standard deviations below that of their own group mean.

Participants were not tested if they had a history of psychiatric or neurological illness. All of the participants possessed normal, or corrected-to-normal visual acuity. Older participants did not report any eye or retinal problems (e.g., macular degeneration, glaucoma, cataracts, etc.), and travelled independently to the site of the experiment. In all experiments, adults provided informed consent and, prior to participating in the study, children’s legal guardians provided written informed consent. All the experimental procedures complied with the protocol approved by the Carnegie Mellon University Internal Review Board.

### Stimuli

Stimuli were 71 pairs of grayscale line-drawings of possible and impossible objects, all of which have been used in previous studies^14, 19^. For each possible object, an impossible object was derived by altering one or a few features, resulting in a modification of the object’s global structure from possible to impossible (Fig. 1). There were no obvious differences in image statistics of the possible and impossible classes as demonstrated by comparisons showing equivalence in the overall number of pixels [37,323 vs. 37,365 for possible vs. impossible objects, respectively] and the number of pixels that defined the object’s edges [3738 vs. 3730 for possible vs. impossible objects, respectively]. Finally, the average pixel-wise correlation between matched possible and impossible objects was r = 0.96, while the average correlation of the non-matched objects was r = 0.22, further reflecting the high visual similarity between the matched objects.

### Procedure

#### General Procedure

Testing took place at the participants’ home (children), at Carnegie Mellon University (older adults, younger adults) or at a local community center (older adults). Stimuli were presented using the E-prime 2.0 software (Psychology Software Tools, Inc., Pittsburgh, PA, USA) and projected on a 14 inch laptop screen (Lenovo, T450s). In all experiments, an example was presented initially to ensure that participants understood the instructions of the specific task. In addition, practice was given prior to the experimental trials and feedback was provided. During the experiment, trials were self-initiated and no feedback was provided. Participants responded using the computer keyboard: a green sticker was stuck on the letter K (‘same’ – Experiment 1 and 3; ‘green is closer’ Experiment 2) and a red sticker stuck on to the letter F (‘different’ - Experiment 1 and 3; ‘red is closer’ - Experiment 2).

#### Experiment 1- Same/different fine-grained classification – matched objects

Experiment 1 was designed to test whether fine-grained 3D representation is automatically derived, even when irrelevant to the task at hand. Participants made speeded same/different judgments on displays in which two objects were presented sequentially. Half of the displays (40 trials) contained pairs of the same object (possible or impossible) and the remaining half (40 trials) contained different objects. Critically, to enforce fine-detailed processing, the ‘different trials’ include a possible object paired with a matched impossible object that differed from the possible object by just a few features (i.e. changes in one or two edges of the object) (Fig. 1b).

On each trial, the first object was presented for 1000 ms, followed by a mask (500 ms) that was composed of scrambled (400 fragments) objects. The second object was then presented until the participant provided a response.

To the extent that 3D structural information is processed in the course of deriving a precise representation of the input, better performance is predicted for trials in which a possible object was presented as the first object compared to trials in which the first object was impossible and a coherent representation could not be generated easily^14, 19, 20^. In addition to the standard instructions (see above), children in the instructed depth group were told that ‘‘the objects you will see are similar to each other but sometimes they may differ because some parts of the objects look further away or closer to you. If the objects look different in their depth - how near or far parts are - you should press the ‘different’ button but if the parts look the same distance from you, you should press the ‘same’ button.’’

#### Experiment 2- Depth comparison of spatial information

The aim of Experiment 2 was to examine whether 3D representations could be derived in children and older adults when 3D information was explicitly processed. One green and one red dot were superimposed on a single object (possible or impossible) that was displayed on the computer screen. Participants were required to judge which of the two dots was located closer in depth by pressing the green or red button^22^ (Fig. 1c). The assignment of the dots was done based on the following logic. First, the dots were located on different surfaces to encourage the processing of the object as a whole. Second, the dots were located on the same position for the possible and matched impossible objects, to maximize the similarity between object categories. Third, the dots were not placed on the junctions the induce object impossibility, to allow an objective “correct” decision in the depth task for the two object categories. Finally, the assignment of the dots was counterbalanced across stimuli such that, for half of the stimuli, the closer dot was lower in the vertical plane and, for the other half, the closer dot was higher in the vertical plane and on half the trials the closer dot was red and on the other half, it was green.

This experiment required the derivation of a precise 3D structural description of the objects. Participant agreement about the close/far spatial location of the dots was validated in a prior pilot study, in which 8 participants (21–43 years old) preformed the task under unconstrained time limitation. High agreement of classification (Possible, 98.1%, SE = 0.5%; Impossible 96.8% SE = 1%) was achieved in this pilot experiment, which reflected the reliability of the perceptual measure used in the current task. Yet, even in the pilot experiment, an advantage was found for possible objects, that were classified faster compared to impossible objects [t_(7)_ = 2.71, p < 0.05]. The experiment included 142 stimuli (half possible and half impossible) that were randomly presented. Each stimulus was presented for an unlimited duration until the participant responded.

For the youngest group of children, an alternative form of Experiment 2 was designed with only 42 trials. Because not all the children in this age were sufficiently trained in using a computer keyboard, the children responded verbally (“red”/“green”) and the experimenter pushed the button for them. Hence, the analysis was restricted to the d′ data. Finally, to ensure that the children were attending to the stimuli and not responding randomly, we included six catch trials in which two blue dots were superimposed on the stimulus, and for these trials, the participant was required to respond that there was neither a red nor green dot in the display. All children successfully identified the catch trials.

#### Experiment 3- Same/different fine-grained classification – non-matched objects

Experiment 3 serves as a control experiment that was designed to rule out an alternative account according to which the observed differences between Experiments 1 and 2 could be related to the different task demands, and not to the degree to which fine-grained depth processing was needed. The procedure was similar to Experiment 1, however the “different” trials consisted of two objects, that were obviously different from each other. The two objects, in each “different” trial, were assigned as a pair based on a pseudo-randomized selection (similar across all participants). Importantly, since “different” trials are so easily discriminable as being different, there is no need to compute fine-grained 3D structural representations of the objects.

### Data analysis

For each condition, RTs longer or shorter than 2.5 standard deviations from the participant mean were excluded from further analysis (3.1% of correct trials, no differences between the three experiments or groups), and were not replaced by other values. Sensitivity scores were adjusted for classification designs^37^:

$$d^{\prime} =2z[\frac{1}{2}\{1+{[2p(c)-1]}^{\frac{1}{2}}\}]$$

where p(c) stands for proportion of correct decision, and z is the inverse of the normal distribution function. Note that we also calculated the d’ based on the more common equation (i.e., *d*′ = *z*(*hit*) − *z*(*false alarms*)) and the results reported along the manuscript were fully. reproduced.

Similar to previous developmental studies^9, 25, 38^, the main dependent variable was the inverse efficiency scores (IE) that were calculated by dividing the Reaction Time (RT) by accuracy scores separately for each condition and participant^39^. The IE allows us to merge RT and accuracy into a single measure to provide a basis for processing efficiency^21^, and to directly compare between groups that usually exhibit effect in terms of RTs (e.g., young adults) to groups that tend to exhibit the behavioral effect in term of accuracy (e.g., children). Note that RT and d′ data are also reported in the manuscript.

Prior to the main between-group analysis, the data were subjected to the Bartlett test of homogeneity of variance^40^. Since this test yielded significant results for IE scores and RT data of Experiment 1 (i.e., the groups have different variances, p < 0.05), we transformed the data so as to ensure a normal distribution. The IE scores and RT data were transformed by taking the log10 of the mean score of each participant and each condition^41^. Parametric analyses (repeated measures ANOVA with group and Experiment as between-subject variables) were then conducted using the transformed dependent measures.

To compare the younger and older children in the depth task, we equalized the number of stimuli. We analyzed the data from the older children based only on the first 42 trials. Since objects were randomly presented, we could not ensure that the number of possible/impossible objects for the first trials subset was equal. However, item analysis validated that, on average, similar numbers of possible and impossible objects were presented (Possible: 21.12, SD: 3.14; Impossible 20.87, SD: 3.14).

## References

[1] Cox DD (2014). Do we understand high-level vision?. Curr. Opin. Neurobiol..

[2] Norcia, A. M. & Gerhard, H. E. Development of Three-Dimensional Perception in Human Infants. *Annu. Rev. Vis. Sci*. 569–594 (2015). 10.1146/annurev-vision-082114-035835 28532379

[3] Soska KC, Johnson SP (2008). Development of Three-Dimensional Object Completion in Infancy. Child Dev..

[4] Soska KC, Adolph KE, Johnson SP (2010). Systems in development: Motor skill acquisition facilitates three-dimensional object completion. Dev. Psychol..

[5] Freud E, Avidan G, Ganel T (2015). The highs and lows of object impossibility: effects of spatial frequency on holistic processing of impossible objects. Psychon. Bull. Rev..

[6] Freud E, Avidan G, Ganel T (2013). Holistic Processing of Impossible Objects: Evidence from Garner’s speeded-classification task. Vision Res..

[7] Kovács I (2000). Human development of perceptual organization. Vision Res..

[8] Kovacs I, Kozma P, Feher A, Benedek G (1999). Late maturation of visual spatial integration in humans. Proc. Natl. Acad. Sci..

[9] Nishimura M, Scherf KS, Zachariou V, Tarr MJ, Behrmann M (2015). Size Precedes View: Developmental Emergence of Invariant Object Representations in Lateral Occipital Complex. J. Cogn. Neurosci..

[10] Andersen GJ (2012). Aging and vision: changes in function and performance from optics to perception. Wiley Interdiscip. Rev. Cogn. Sci..

[11] Norman JF, Crabtree CE, Bartholomew AN, Ferrell EL (2009). Aging and the perception of slant from optical texture, motion parallax, and binocular disparity. Percept. Psychophys..

[12] Norman JF, Bartholomew AN, Burton CL (2008). Aging preserves the ability to perceive 3D object shape from static but not deforming boundary contours. Acta Psychol. (Amst.).

[13] Holmin J, Nawrot M (2016). The effects of aging on the perception of depth from motion parallax. Atten. Percept. Psychophys..

[14] Freud E, Ganel T, Avidan G (2015). Impossible expectations: fMRI adaptation in the lateral occipital complex (LOC) is modulated by the statistical regularities of 3D structural information. NeuroImage.

[15] Shuwairi SM, Albert MK, Johnson SP (2007). Discrimination of possible and impossible objects in infancy. Psychol Sci.

[16] Kavšek M, Yonas A, Granrud CE (2012). Infants’ sensitivity to pictorial depth cues: A review and meta-analysis of looking studies. Infant Behav. Dev..

[17] Enns JT, Girgus JS (1986). A developmental study of shape integration over space and time. Dev. Psychol..

[18] Schacter DL, Cooper LA, Valdiserri M (1992). Implicit and explicit memory for novel visual objects in older and younger adults. Psychol. Aging.

[19] Freud, E. *et al*. Three-Dimensional Representations of Objects in Dorsal Cortex are Dissociable from Those in Ventral Cortex. *Cereb. Cortex* bhv229, doi:10.1093/cercor/bhv229 (2015). 10.1093/cercor/bhv229 PMC13100970 26483400

[20] Schacter DL, Cooper LA, Delaney SM (1990). Implicit memory for unfamiliar objects depends on access to structural descriptions. J Exp Psychol Gen.

[21] Townsend JT, Ashby FG (1978). Methods of modeling capacity in simple processing systems. Cogn. Theory.

[22] Koenderink JJ, van Doorn AJ, Kappers AM (1996). Pictorial surface attitude and local depth comparisons. Percept Psychophys.

[23] Salthouse TA (1996). The processing-speed theory of adult age differences in cognition. Psychol. Rev..

[24] Kail R (1991). Developmental change in speed of processing during childhood and adolescence. Psychol. Bull..

[25] Yang S, Yang H, Lust B (2011). Early childhood bilingualism leads to advances in executive attention: Dissociating culture and language. Biling. Lang. Cogn..

[26] Nava E, Rinaldi L, Bulf H, Cassia VM (2017). Visual and proprioceptive feedback differently modulate the spatial representation of number and time in children. J. Exp. Child Psychol..

[27] Goffaux V, Hault B, Michel C, Vuong QC, Rossion B (2005). The respective role of low and high spatial frequencies in supporting configural and featural processing of faces. Perception.

[28] Akhtar N, Enns JT (1989). Relations between convert orienting and filtering in the development of visual attention. J. Exp. Child Psychol..

[29] Bruyer R, Brysbaert M (2011). Combining speed and accuracy in cognitive psychology: is the inverse efficiency score (IES) a better dependent variable than the mean reaction time (RT) and the percentage of errors (PE)?. Psychol. Belg..

[30] Simion F, Leo I, Turati C, Valenza E, Dalla Barba B (2007). How face specialization emerges in the first months of life. Prog. Brain Res..

[31] de Heering A (2008). Newborns’ face recognition is based on spatial frequencies below 0.5 cycles per degree. Cognition.

[32] Johnson, M. H. & Morton, J. *Biology and cognitive development: The case of face recognition*. (Blackwell, 1991).

[33] Johnson MH (2005). Subcortical face processing. Nat. Rev. Neurosci..

[34] Mondloch CJ, Dobson KS, Parsons J, Maurer D (2004). Why 8-year-olds cannot tell the difference between Steve Martin and Paul Newman: Factors contributing to the slow development of sensitivity to the spacing of facial features. J. Exp. Child Psychol..

[35] Germine LT, Duchaine B, Nakayama K (2011). Where cognitive development and aging meet: Face learning ability peaks after age 30. Cognition.

[36] Elman JL (1993). Learning and development in neural networks: The importance of starting small. Cognition.

[37] Macmillan, N. A. & Creelman, C. D. *Detection theory: A user’s guide*. (Psychology press, 2004).

[38] Iuculano T, Tang J, Hall CW, Butterworth B (2008). Core information processing deficits in developmental dyscalculia and low numeracy. Dev. Sci..

[39] Townsend, J. T. & Ashby, F. G. *Stochastic modeling of elementary psychological processes*. (Cambridge: Cambridge University Press., 1983).

[40] Snedecor, G. W. & Cochran, W. G. *Statistical Methods*. (Iowa State University Press., 1989).

[41] McDonald, J. H. *Handbook of Biological Statistics*. (Sparky House Publishing, 2014).

